# Supplementary material for: Synergistic Action of Gefitinib and GSK41364A Simultaneously Loaded in Ratiometrically-Engineered Polymeric Nanoparticles for Glioblastoma Multiforme
Source: J Clin Med. 2019 Mar 15;8(3):367. doi: 10.3390/jcm8030367 (PMC6462915; doi:10.3390/jcm8030367)
Supplement: Supplementary file 1 [file jcm-08-00367-s001.pdf]

Supplementary Materials:

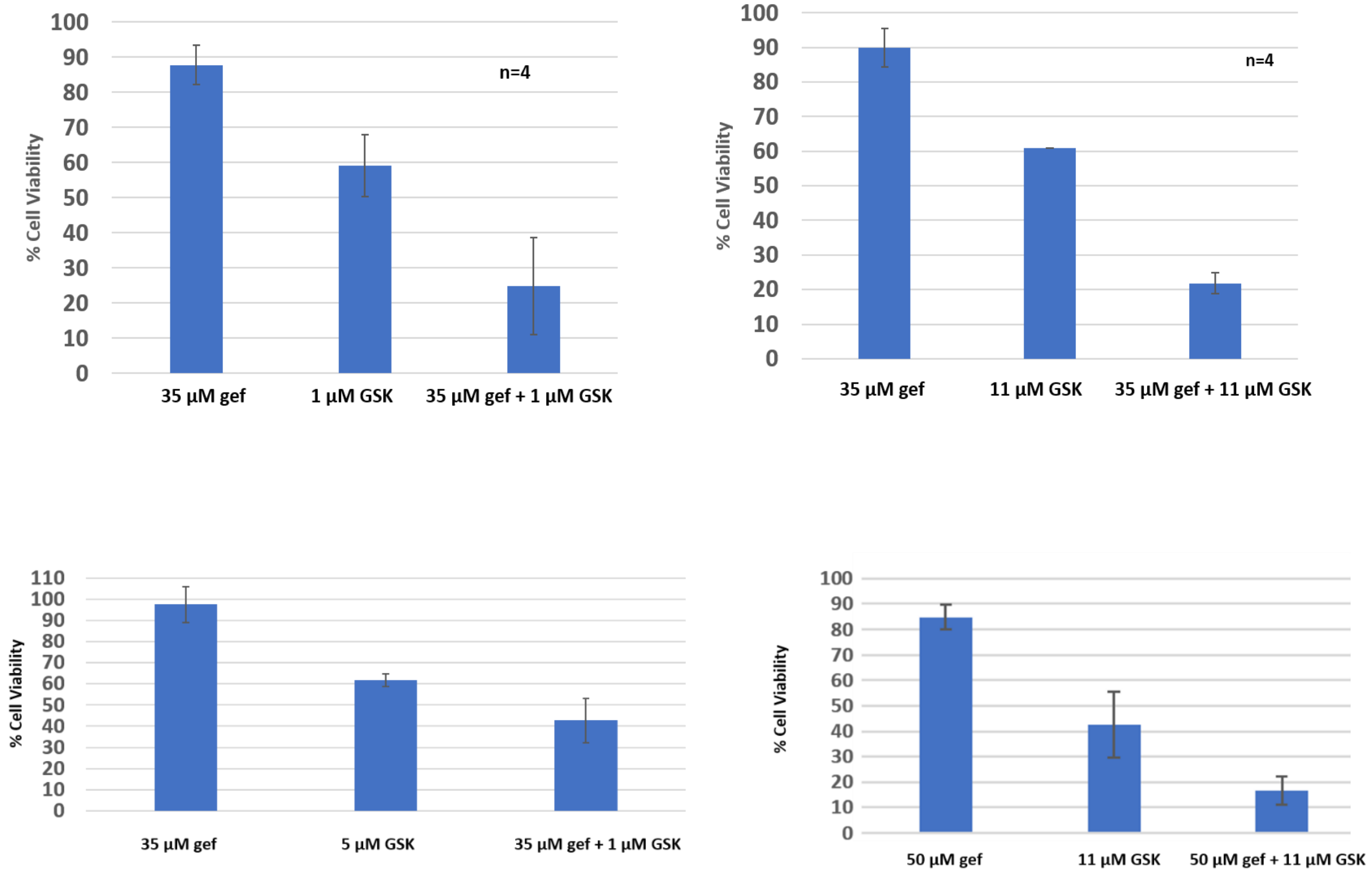

Supplementary Figure S1: Evaluation of cell viability post treatment with different sets of combination of free gefitinib and free GSK461364A

(2A)

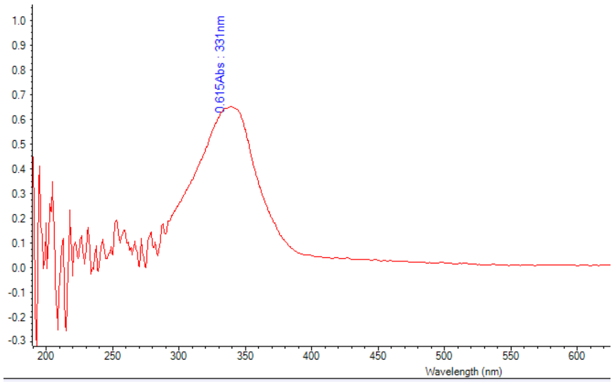

(2B)

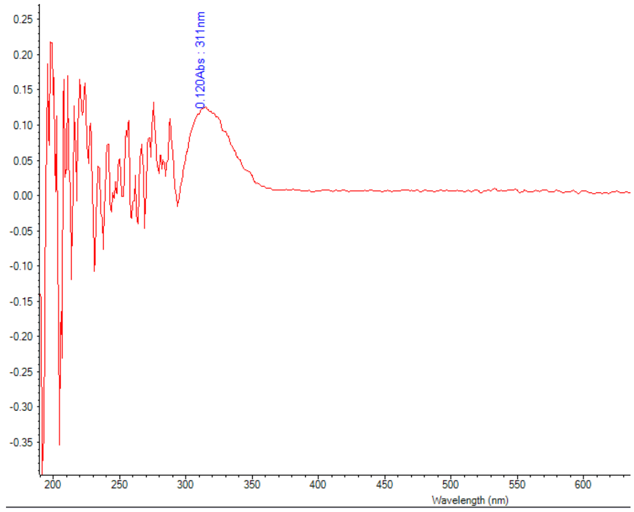

Supplementary Figure S2 : Peak absorbance of **(2A)** gefitinib at 331nm dissolved in 1% Triton-x, with GSK461364A as blank **(2B)** GSK461364A at 311nm with gefitinib as blank

(3A)

			Size (d.nm):	% Intensity:	St Dev (d.nm):
Z-Average (d.nm):	122.1	Peak 1:	135.5	100.0	43.99
PdI:	0.091	Peak 2:	0.000	0.0	0.000
Intercept:	0.966	Peak 3:	0.000	0.0	0.000
Result quality :	Good				

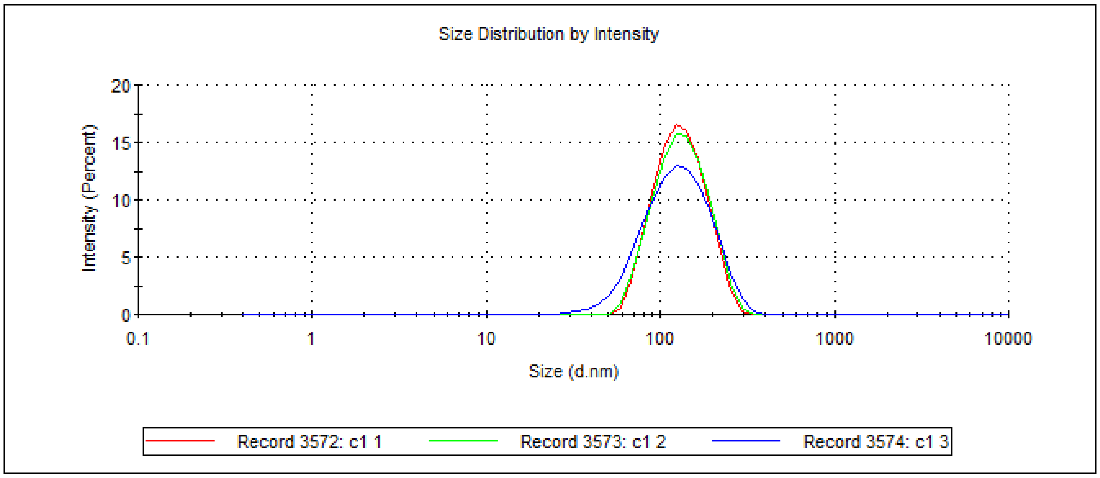

(3B)

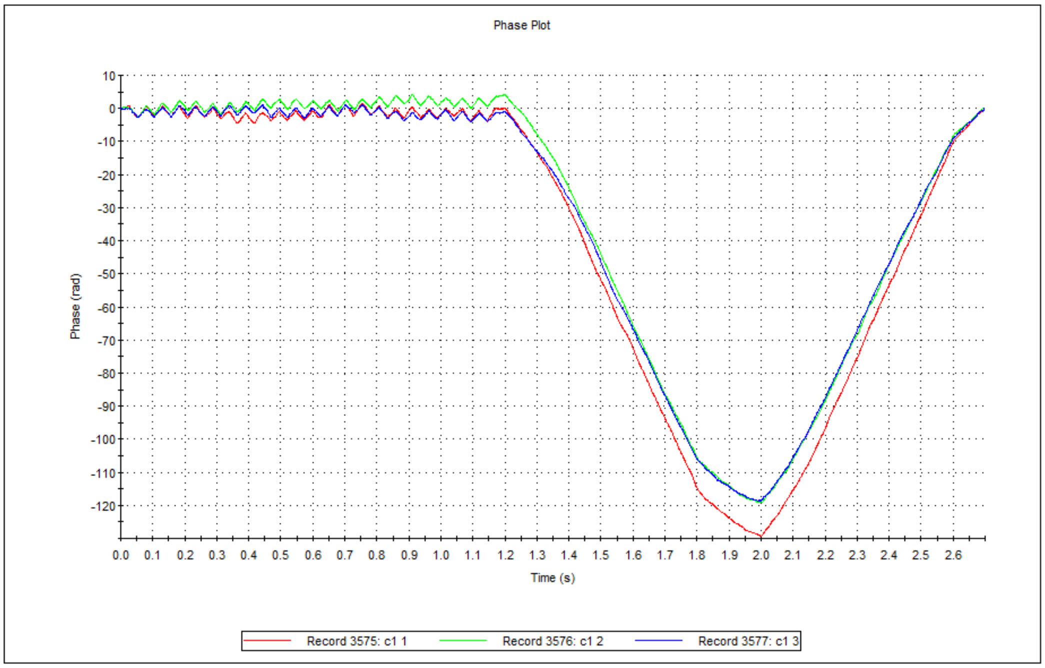

(3C)

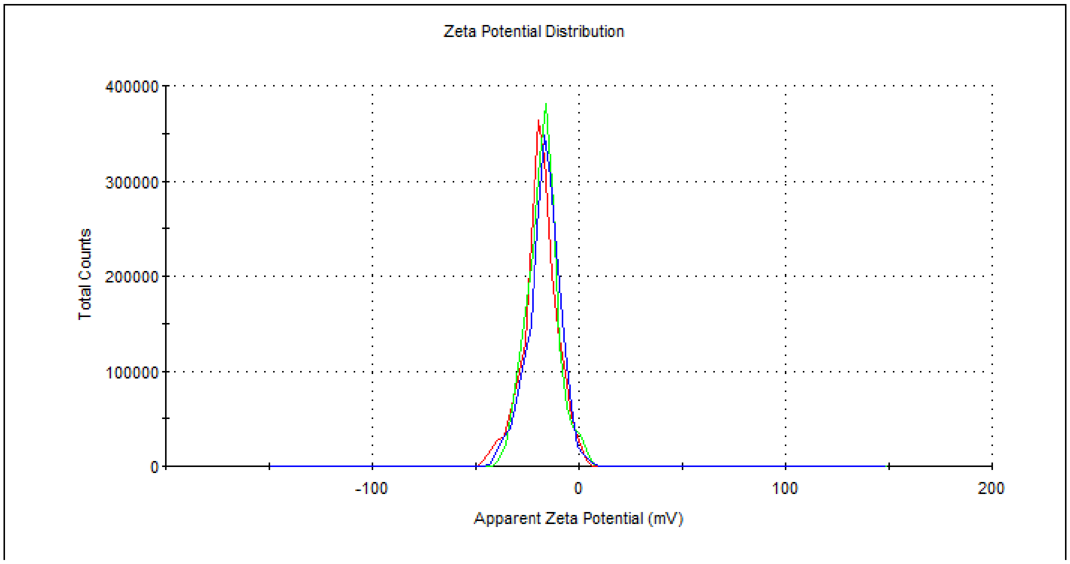

Supplementary Figure S3: (A) Size distribution by intensity with the average size being ~122 nm. (B) Phase plot of zeta potential plotted against time (C) Zeta potential distribution with total counts plotted against apparent zeta potential. Average zeta potential was ~20 mV.

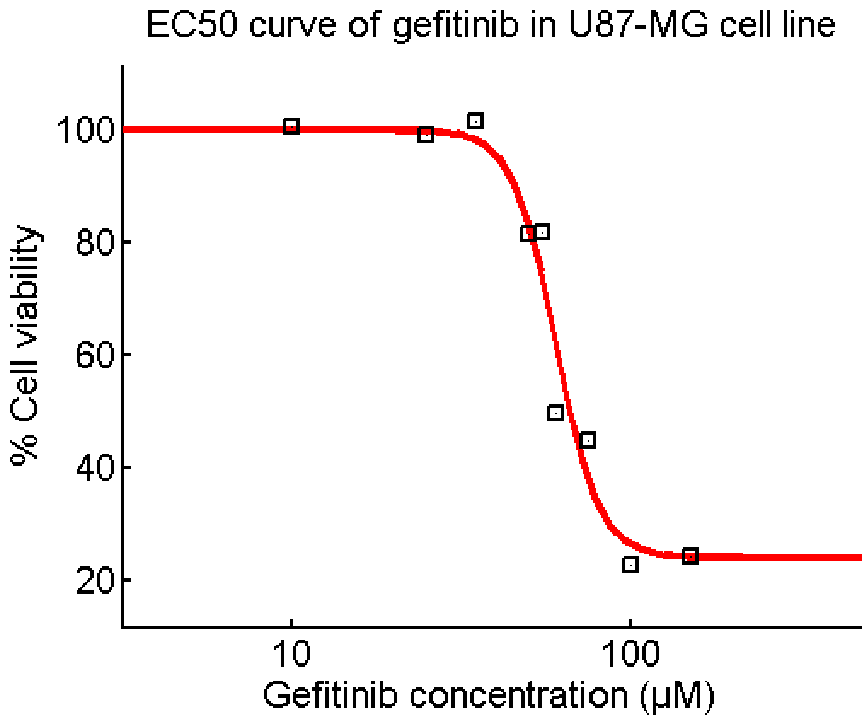

Supplementary Figure S4: Effective concentration (EC_{50}) curve of gefitinib in U87-MG cell line plotted using Dr.Fit© software. These data points follow monophasic model (standard Hill equation).
